# Is neoadjuvant immunotherapy feasible for patients with dMMR/MSI−H locally advanced colorectal cancer? a retrospective study

**DOI:** 10.3389/fimmu.2025.1645412

**Published:** 2025-08-12

**Authors:** Zhenkai Luo, Danjun Wang, Zhexue Wang, Hang Zhao, Renshen Xiang

**Affiliations:** ^1^ Department of Endoscopy, Zhejiang Cancer Hospital, Hangzhou Institute of Medicine (HIM), Chinese Academy of Sciences, Hangzhou, China; ^2^ Department of Anesthesiology, Shanghai Geriatric Medical Center, Shanghai, China; ^3^ Department of Colorectal Surgery, National Cancer Center/National Clinical Research Center for Cancer/Cancer Hospital, Chinese Academy of Medical Sciences & Peking Union Medical College, Beijing, China; ^4^ Department of Head and Neck Surgery, Fudan University Shanghai Cancer Center, Shanghai, China; ^5^ Department of Oncology, Shanghai Medical College, Fudan University, Shanghai, China; ^6^ Tianjin’s Clinical Research Center for Cancer, Tianjin Key Laboratory of Digestive Cancer, Tianjin Medical University Cancer Institute & Hospital, National Clinical Research Center for Cancer, Tianjin, China

**Keywords:** locally advanced colorectal cancer, immunotherapy, neoadjuvant therapy, microsatellite instability, pathological complete response

## Abstract

**Background:**

Neoadjuvant immunotherapy has demonstrated satisfactory efficacy for high microsatellite instability/mismatch repair deficiency (dMMR/MSI-H) in locally advanced colorectal cancer (LACRC). This study aims to evaluate the safety and short-term efficacy of neoadjuvant immunotherapy for patients with LACRC.

**Methods:**

We retrospectively analyzed patients with dMMR/MSI-H LACRC who received neoadjuvant immunotherapy at two Chinese medical centers. The primary outcome of the study was the pathological complete response (pCR) rate, while secondary endpoints included survival status, perioperative outcomes and safety profile.

**Results:**

A total of 26 patients were included in the analysis, with a median age of 58 years (range: 33–78 years). All patients underwent radical surgery after completing neoadjuvant immunotherapy. All patients achieved R0 resection, and the pCR rate was 92.3% (24/26). Furthermore, all patients experienced downstaging (100%). Eight patients (30.8%) experienced immune-related adverse events (IRAEs), and five patients (19.2%) developed postoperative complications. The median follow-up duration was 19.0 months (range: 4.0-41.0 months). No patients died during the follow-up period, and no local recurrence or distant metastasis was observed.

**Conclusion:**

Neoadjuvant immunotherapy appears to be a safe and effective treatment for patients with dMMR/MSI-H LACRC, offering a feasible and acceptable therapeutic regimen before surgery.

## Introduction

Colorectal cancer (CRC) is one of the most common and fatal malignant tumors globally, ranking second in incidence in China (1). The majority of patients are diagnosed at advanced or late stages, leading to poor treatment outcomes and prognosis. Locally advanced colorectal cancer (LACRC) refers to CRC at clinical stages II or III (i.e., cT3-4N0 or any T, N+) (2). In recent years, advances in standardized surgery and the development of neoadjuvant therapies have significantly improved the survival outcomes of LACRC patients. Neoadjuvant therapy can downstage tumors, increase the rate of R0 resection, reduce local recurrence, and allow some patients to achieve clinical complete response (cCR) or even pathological complete response (pCR) (3, 4). In cases of metastasis, chemotherapy remains the primary treatment modality. CRC is primarily caused by genomic instability resulting from the accumulation of oncogene mutations or tumor suppressor gene inactivation. Approximately 10%-15% of sporadic CRC cases exhibit mismatch repair deficiency (dMMR) or high microsatellite instability (MSI-H) (5). In 2015, the KEYNOTE-016 study (NCT01876511) demonstrated that dMMR/MSI-H metastatic CRC (mCRC) patients significantly benefit from programmed cell death protein-1 (PD-1) inhibitors, marking the onset of the immunotherapy era for CRC (6).

Currently, immunotherapy is recommended by guidelines as the first-line treatment for dMMR/MSI-H mCRC. However, dMMR/MSI-H CRC accounts for only 5% of stage IV CRC cases, limiting the patient population eligible for immunotherapy. This proportion increases to up to 15% in stage II and III patients (7). Consequently, recent efforts have focused on expanding the eligible population for immunotherapy. For dMMR/MSI-H LACRC, several completed or ongoing clinical trials have investigated the efficacy of neoadjuvant immunotherapy, either as monotherapy or in combination with chemoradiotherapy (8, 9). Some of these trials, such as the NICHE study, have yielded promising results, including a pathological response rate exceeding 95%, a pCR rate of 69%, and a 13-month recurrence-free survival (RFS) rate of 100% (10).

These findings have increased confidence in the potential of immunotherapy for CRC. However, current guidelines do not explicitly recommend immunotherapy as a preoperative treatment for LACRC, and related clinical studies are still ongoing. The efficacy and safety of neoadjuvant immunotherapy, including its impact on surgical difficulty and potential complications, remain key concerns in clinical practice. Therefore, this retrospective study aims to evaluate the safety and short-term efficacy of neoadjuvant immunotherapy in patients with LACRC, providing valuable guidance and reference for clinical decision-making.

## Methods

### Patient selection

This retrospective study was approved by the Ethics Committees of Zhejiang Cancer Hospital and Cancer Hospital, Chinese Academy of Medical Sciences. Due to the retrospective nature of the study, informed consent was waived. We retrospectively collected data from patients diagnosed with CRC who received preoperative PD-1/programmed death-ligand 1 (PD-L1) blockade (envafolimab, tislelizumab, sintilimab, pembrolizumab and nivolumab) immunotherapy, with or without chemotherapy, at the two hospitals between January 2019 and January 2025. All patients underwent pretreatment clinical staging through standardized imaging protocols including chest-abdomen-pelvis computed tomography (CT), magnetic resonance imaging (MRI), and positronemission tomography (PET). The inclusion criteria were: (1) pathologically confirmed rectal adenocarcinoma; (2) colon or high rectal cancer (≥10 cm from the anus) at clinical stage II or III; (3) dMMR/MSI-H status; and (4) an Eastern Cooperative Oncology Group (ECOG) performance status of 0 or 1. The exclusion criteria included: (1) patients under the age of 18; (2) a history of prior immunotherapy or immune diseases; and (3) patients who did not undergo surgical treatment.

### Data collection and follow-up

Demographic, operative, and clinical data were collected for analysis. Patients were monitored every 3 months for the first 2 years post-surgery and every 6 months thereafter for up to 5 years. Follow-up assessments included laboratory tests, imaging examinations, and colonoscopy. Overall survival (OS) was defined as the time from the date of surgery to death from any cause. Disease-free survival (DFS) was defined as the time from surgery to disease recurrence or death from any cause.

### Pathological evaluation

All resected tumor specimens were sent for pathological examination by senior pathologists. Pathological staging was performed according to the neoadjuvant pathological staging (ypTNM) system as per the eighth edition of the American Joint Committee on Cancer (AJCC) (11). R0 resection was defined as resection with a microscopic negative margin. The pCR was defined as the complete disappearance of tumor tissue, with no residual tumor cells found in the tumor tissue or lymph nodes following surgery. Major pathological response (MPR) was defined as the presence of residual tumor ≤10% after assessment of the primary lesion (12).

### Efficacy and safety assessment

Response to treatment was systematically assessed by chest-abdomen-pelvis CT, MRI, serum carcinoembryonic antigen (CEA), endoscopy, and selective biopsy of any residual mass or scar. Regional lymph nodes were defined as metastatic if they demonstrated either a short-axis diameter >10 mm with round morphology, or a short-axis diameter of 5–9 mm meeting at least two of the following criteria: round shape, irregular borders, or heterogeneous signal intensity on CT/MRI, with PET imaging utilized for further evaluation in diagnostically challenging cases. Tumor response was evaluated after every 2 to 3 cycles of anti–PD-1/PD-L1 immunotherapy according to the revised RECIST guidelines (version 1.1) (13). Tumor regression grading (TRG) assessment was performed using the 8th edition of the AJCC guidelines, which classifies tumor regression into four grades, with lower grades indicating more pronounced tumor regression (11). Treatment-related adverse events (TRAEs) were graded according to the Common Terminology Criteria for Adverse Events (CTCAE, version 5.0). Among these, immune-related adverse events (IRAEs) were specifically recorded. Postoperative complications were classified using the Clavien-Dindo classification (14).

### Statistical analysis

Categorical variables were expressed as frequencies (percentages). Continuous variables were presented as median values with ranges. Survival analysis was performed using the Kaplan-Meier method. All statistical analyses and figure generation were conducted using R software (Version 4.1.2).

## Results

### Patient characteristics

A total of 26 patients were enrolled based on the inclusion and exclusion criteria. The median age of the patients was 58 years (range: 33–78 years). Of these, 8 patients were diagnosed at clinical stage II, while 18 were at clinical stage III. Twelve patients had clinical T3 tumors, and 14 had clinical T4 tumors. Eight patients were at clinical N0 stage, and 18 were at clinical N+ stage. Seven patients had family histories of Lynch syndrome. All patients were confirmed to have dMMR through immunohistochemistry (IHC). Some patients had their microsatellite instability (MSI) status assessed via PCR and next-generation sequencing (NGS). Seventeen patients received PD-1/PD-L1 blockade monotherapy as neoadjuvant therapy, and 9 patients received a combination of PD-1/PD-L1 blockade and chemotherapy. Detailed characteristics are presented in **Tables 1**, **2**.

**Table 1:** Patient baseline characteristics (N=26).

Characteristic	n (%)
Age, median (range), years	58 (33-78)
Sex	
Male	17 (65.4)
Female	9 (34.6)
ECOG performance status score	
0	21 (80.8)
1	5 (19.2)
Clinical T stage	
T3	13 (46.2)
T4	13 (53.8)
Clinical N stage	
N0	8 (30.8)
N+	18 (69.2)
Tumor site	
Colon	24 (92.3)
Rectum	2 (7.7)
Lynch syndrome	
Yes	7 (26.9)
No	19 (73.1)
Mismatch repair status	
MLH1 or PMS2 deficient, or both	16 (61.5)
MSH2 or MSH6 deficient, or both	10 (38.5)

ECOG, Eastern Cooperative Oncology Group.

**Table 2:** Details of the 26 patients with neoadjuvant ICB therapy.

Patient	Sex	Age	Tumor location	Clinical TNM stage	Invaded organ	ICB	Courses of ICB	Neoadjuvant chemotheropy	Tumor response	Surgery	Pathological stage	TRG
1	Male	60	Colon	cT4bN1M0	Adjacent colon	Envafolimab	8	NA	MPR	CME	ypT0N1	1
2	Female	78	Colon	cT4bN1M0	Bladder	Tislelizumab	1	NA	PR	CME+partial cystectomy	ypT3N0	2
3	Male	47	Colon	cT4aN1M0	NA	Sintilimab	3	NA	pCR	CME	ypT0N0	0
4	Male	58	Colon	cT4aN0M0	NA	Pembrolizumab	3	NA	pCR	CME	ypT0N0	0
5	Male	66	Colon	cT4bN0M0	Small intestine	Tislelizumab	3	NA	pCR	CME+partial small bowel resection	ypT0N0	0
6	Female	69	Colon	cT3N0M0	NA	Tislelizumab	3	NA	pCR	CME	ypT0N0	0
7	Male	49	Colon	cT4aN0M0	NA	Tislelizumab	3	NA	pCR	CME	ypT0N0	0
8	Female	37	Colon	cT4aN1M0	NA	Pembrolizumab	6	NA	pCR	CME	ypT0N0	0
9	Male	74	Rectum	cT3N0M0	NA	Envafolimab	2	XELOX × 2	pCR	PME	ypT0N0	0
10	Female	41	Colon	cT3N2M0	NA	Tislelizumab	6	XELOX × 6	pCR	CME	ypT0N0	0
11	Male	37	Colon	cT3N0M0	NA	Nivolumab	6	XELOX × 2 Pre ICB	pCR	CME	ypT0N0	0
12	Female	58	Colon	cT3N2M0	NA	Envafolimab	4	NA	pCR	CME	ypT0N0	0
13	Male	46	Colon	cT3N2M0	NA	Sintilimab	2	XELOX × 1 Pre ICB	pCR	CME	ypT0N0	0
14	Male	74	Colon	cT3N2M0	NA	Sintilimab	7	XELOX × 3 Pre ICB	pCR	CME	ypT0N0	0
15	Female	43	Colon	cT4aN2M0	NA	Tislelizumab	4	NA	pCR	CME	ypT0N0	0
16	Male	61	Colon	cT4N1M0	NA	Tislelizumab	3	NA	pCR	CME	ypT0N0	0
17	Male	37	Colon	cT4aN1M0	NA	Tislelizumab	4	XELOX × 4	pCR	CME	ypT0N0	0
18	Female	47	Colon	cT4bN1M0	Bladder	Envafolimab	2	XELOX × 2	pCR	CME	ypT0N0	0
19	Female	70	Colon	cT4aN1M0	NA	Tislelizumab	2	NA	pCR	CME	ypT0N0	0
20	Male	49	Colon	cT3N0M0	NA	Pembrolizumab	5	NA	pCR	CME	ypT0N0	0
21	Male	33	Colon	cT3N1M0	NA	Envafolimab	4	NA	pCR	CME	ypT0N0	0
22	Female	75	Colon	cT3N1M0	NA	Tislelizumab	4	NA	pCR	CME	ypT0N0	0
23	Male	64	Colon	cT4bN1M0	Ureter	Sintilimab	4	FOLFOX × 4	pCR	CME	ypT0N0	0
24	Male	60	Rectum	cT3N1M0	NA	Sintilimab	2	FOLFOX × 2	pCR	PME	ypT0N0	0
25	Male	60	Colon	cT4aN1M0	NA	Envafolimab	8	NA	pCR	CME	ypT0N0	0
26	Male	52	Colon	cT3N0M0	NA	Envafolimab	9	NA	pCR	CME	ypT0N0	0

ICB, immune checkpoint blockade; TRG, tumor regression grade; NA, not available; MPR, major pathological response; PR, partial response; pCR, pathological complete response; CME, complete mesocolic excision; PME: partial mesorectal excision.

### Efficacy of neoadjuvant immunotherapy

**Figure 1:**
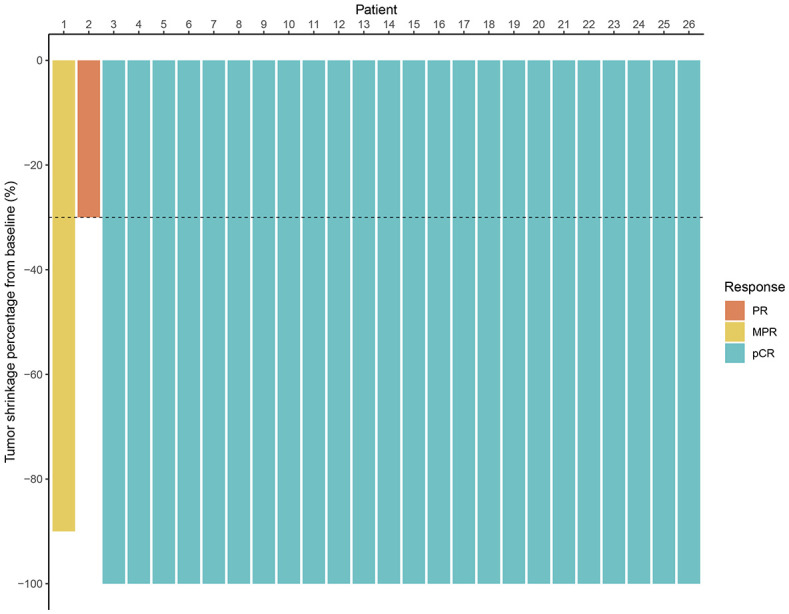
Waterfall plot of efficacy evaluation of neoadjuvant immunotherapy in dMMR/MSI-H LACRC. (MPR, major pathological response; PR, partial response; pCR, pathological complete response).

All patients underwent radical surgery after completing neoadjuvant immunotherapy. Among patients with clinical T4b stage, some required additional partial organ resections based on intraoperative findings (**Table 2**). The median interval between the end of treatment and surgery was 6 weeks (range: 3–13 weeks). The median operative time was 138 minutes (range: 85–260 minutes), and the median postoperative hospital stay was 6 days (range: 4–20 days). According to postoperative pathological diagnoses, all patients achieved R0 resection. The efficacy evaluation of neoadjuvant immunotherapy is presented in **Figure 1**. The pCR rate was 92.3% (24/26), and the MPR rate was 96.2% (25/26). Regarding preoperative clinical staging (T and N staging), all patients experienced downstaging (26/26, 100%). All patients continued immunotherapy after surgery. For those who achieved pCR, treatment was maintained for one year, whereas for non-pCR patients, immunotherapy was extended to two years. The median follow-up duration was 19.0 months (range: 4.0-41.0 months). No patients died during the follow-up period, and no local recurrence or distant metastasis was observed. The 3-year OS rate and DFS rate were both 100%.

### Safety and feasibility

During immunotherapy, 8 patients (30.8%) experienced IRAEs. The most common adverse events were rash (7.7%), gastrointestinal reactions (7.7%), hypothyroidism (3.8%), adrenocortical insufficiency, myocarditis, and elevated aminotransferase levels (3.8%). Among the patients who developed IRAEs, 26.9% (7/26) experienced grade I–II adverse events, while 3.8% (1/26) had grade III–IV events. Patient 2 developed a grade III IRAE after the first cycle of ICB treatment, which resulted in the immediate discontinuation of the treatment. None of these patients underwent surgery earlier than planned. Five patients developed postoperative complications, as detailed in **Table 3**. The overall complication rate was 19.2% (5/26). All patients with postoperative complications were discharged after conservative treatment without requiring secondary surgery, and no perioperative deaths occurred.

**Table 3:** Adverse events.

Adverse Events	Grade I-II (%)	Grade III-IV (%)
Immuno-related (n = 26)		
Rash	2 (7.7)	0 (0)
Hypothyroidism	0 (0)	1 (3.8)
Adrenocortical insufficiency	1 (3.8)	0 (0)
Myocarditis	1 (3.8)	0 (0)
Increased aminotransferase	1 (3.8)	0 (0)
Gastrointestinal reaction	2 (7.7)	0 (0)
Surgery-related (n = 26)		
Anastomotic leak	1 (3.8)	0 (0)
Obstruction/Ileus	2 (7.7)	0 (0)
Infection	1 (3.8)	0 (0)
Bleeding	1 (3.8)	0 (0)

## Discussion

In this study, we evaluated the safety and efficacy of neoadjuvant immunotherapy in patients with dMMR/MSI-H LACRC across two centers. Neoadjuvant radiotherapy or chemoradiotherapy is the standard treatment for locally advanced rectal cancer (LARC) located less than 10 cm from the anal verge. We excluded patients with mid to low rectal cancer to avoid the confounding effects of radiotherapy. Our findings indicate that neoadjuvant immunotherapy is both safe and effective, representing a promising strategy for treating dMMR/MSI-H LACRC.

Given the promising results of immunotherapy in dMMR/MSI-H mCRC, there has been increasing interest in expanding its use to preoperative treatment for locally advanced or even early-stage CRC, potentially benefiting a broader patient population. The NICHE-1 study enrolled 20 patients with dMMR/MSI-H non-metastatic colon cancer who received two cycles of neoadjuvant immunotherapy prior to surgery, resulting in a pCR in 12 patients (60%) (15). The larger NICHE-2 study involved 111 patients with dMMR/MSI-H non-metastatic colon cancer who also received two cycles of neoadjuvant immunotherapy, leading to a pCR in 75 patients (67.6%). With a median follow-up of 26 months, no patients experienced tumor recurrence or metastasis (10). In another study by Cercek et al., 12 patients with stage II and III dMMR/MSI-H rectal cancer who received PD-1 inhibitor therapy every 3 weeks for 6 months achieved a 100% cCR during follow-up (9). Similarly, the PICC study included 34 dMMR/MSI-H LACRC patients, divided into two groups: one group received PD-1 inhibitors combined with celecoxib, achieving a pCR rate of 88% (15/17), while the other group received PD-1 inhibitors alone, achieving a pCR rate of 65% (11/17) (8). In our study, the pCR rate after neoadjuvant immunotherapy in MSI-H LACRC patients was as high as 92.3% (24/26). For patients who did not achieve pCR, substantial tumor regression was observed, likely related to a high tumor burden and the relatively short treatment cycles. These findings collectively demonstrate that neoadjuvant immunotherapy can yield satisfactory results in resectable dMMR/MSI-H CRC.

Currently, the selection of patients who may benefit from immunotherapy relies on testing for MSI or dMMR status prior to treatment. However, not all dMMR/MSI-H CRC patients respond to immunotherapy. In the CheckMate study, 13% of dMMR/MSI-H mCRC patients were resistant to PD-1 inhibitors (16). Furthermore, most CRC patients are not dMMR/MSI-H, and using MSI status as the sole selection criterion excludes a large portion of patients who may not benefit from immunotherapy. Thus, more precise and reliable biomarkers are needed to identify patients who will respond to treatment. Tumor mutational burden (TMB) is one such biomarker. TMB, which measures the number of somatic mutations in a tumor, has been shown to correlate with neoantigen production and immunogenicity, enhancing the efficacy of immunotherapy in microsatellite-stable (MSS) CRC (17–19). Another promising biomarker is POLE/POLD1. POLE mutations, which affect DNA replication and repair, can lead to hypermutated, microsatellite-stable CRCs that share immune characteristics with dMMR/MSI-H tumors (20, 21). In this study, not all patients underwent NGS, and thus, the status of POLE/POLD1 mutations remains unknown for these patients. Patients with POLE-mutated CRCs have shown responses to immune checkpoint inhibitors (ICIs) in case reports (22). The 2024 NCCN guidelines now recommend genetic testing for POLE/OLD1 mutations in patients, emphasizing the importance of screening even MSS patients for potential immunotherapy benefit (23).

While immunotherapy has demonstrated encouraging results, its safety profile remains a subject of concern, particularly regarding IRAEs. The mechanisms underlying IRAEs are not fully understood, but they can affect multiple organs, with the skin, gastrointestinal tract, liver, and endocrine system being the most commonly affected (24, 25). In the KEYNOTE-177 study targeting dMMR/MSI-H mCRC, 31% of patients in the pembrolizumab group experienced IRAEs, with 9% of these being grade III or IV, including colitis and hepatitis (26). This may be associated with factors including high tumor burden, extended immunotherapy duration, and poor patient performance status. Emerging safety profiles from neoadjuvant immunotherapy trials have also been increasingly reported in recent years. In the NICHE-1 study, 23 patients (13%) experienced grade I-II IRAEs, while 5 patients (13%) developed grade III-IV IRAEs (15). Moreover, in a neoadjuvant PD-1 inhibitor study for LARC, IRAEs occurred in 53% of patients (9/17), with only one grade III IRAE observed (6%) (27). Although our study observed an overall incidence of IRAEs of 30.8%, with only one patient (3.8%) experiencing a grade III IRAE, which suggests that the safety profile appears acceptable, further research is required to fully elucidate these findings.

A key concern with neoadjuvant immunotherapy is its potential impact on surgery and perioperative safety. IRAEs may delay surgery or lead to tissue edema, fibrosis, and increased bleeding at the surgical site, thereby complicating surgery and increasing the risk of postoperative complications (28). In the NICHE study, 20% of patients undergoing colon resection experienced grade III surgical complications, including a 10% anastomotic leak rate, which may have been influenced by the combined use of PD-1 and Cytotoxic T-lymphocyte antigen 4 (CTLA-4) inhibitors (15). In our study, all patients underwent radical R0 resection, and no significant increase in surgical time or blood loss was observed. The overall postoperative complication rate was 19.2%, which is consistent with the range seen in conventional colorectal cancer surgeries (10%-37%) (29). No significant increase in anastomotic leaks was noted, and there were no perioperative deaths. These results suggest that neoadjuvant immunotherapy does not significantly increase surgical risks or postoperative complications. However, careful perioperative management, thorough preoperative assessments, and meticulous surgical procedures are crucial for optimal outcomes.

Our study has several limitations. First, its retrospective nature and small sample size introduce inherent bias. Second, patients in this study received various PD-1/PD-L1 inhibitors and were treated across different therapeutic cycles, with some also undergoing chemotherapy. This diversity in treatment regimens complicates the interpretation of our findings. Lastly, this study was conducted in a Chinese population, and the findings may not be universally applicable. Future large-scale, more standardized multicenter studies with extended follow-up are needed to better confirm the clinical benefits of neoadjuvant immunotherapy for LACRC.

## Conclusion

Neoadjuvant immunotherapy appears to be a safe and effective treatment for patients with dMMR/MSI-H LACRC, offering a feasible and acceptable therapeutic regimen before surgery. However, large-scale prospective studies are required to further confirm these findings.

## Data Availability

The raw data supporting the conclusions of this article will be made available by the authors, without undue reservation.

## References

[1] Xia C Dong X Li H Cao M Sun D He S. Cancer statistics in China and United States, 2022: profiles, trends, and determinants. Chin Med J (Engl). (2022) 135:584–90. doi: 10.1097/CM9.0000000000002108, PMID: 35143424 PMC8920425

[2] Sinicrope FA Chakrabarti S Laurent-Puig P Huebner L Smyrk TC Tabernero J. Prognostic variables in low and high risk stage III colon cancers treated in two adjuvant chemotherapy trials. Eur J Cancer. (2021) 144:101–12. doi: 10.1016/j.ejca.2020.11.016, PMID: 33341444 PMC7855426

[3] Kasi A Abbasi S Handa S Al-Rajabi R Saeed A Baranda J. Total neoadjuvant therapy vs standard therapy in locally advanced rectal cancer: A systematic review and meta-analysis. JAMA Netw Open. (2020) 3:e2030097. doi: 10.1001/jamanetworkopen.2020.30097, PMID: 33326026 PMC7745099

[4] Sauer R Liersch T Merkel S Fietkau R Hohenberger W Hess C. Preoperative versus postoperative chemoradiotherapy for locally advanced rectal cancer: results of the German CAO/ARO/AIO-94 randomized phase III trial after a median follow-up of 11 years. J Clin Oncol. (2012) 30:1926–33. doi: 10.1200/JCO.2011.40.1836, PMID: 22529255

[5] Vilar E Gruber SB. Microsatellite instability in colorectal cancer-the stable evidence. Nat Rev Clin Oncol. (2010) 7:153–62. doi: 10.1038/nrclinonc.2009.237, PMID: 20142816 PMC3427139

[6] Franke AJ Skelton WP Starr JS Parekh H Lee JJ Overman MJ. Immunotherapy for colorectal cancer: A review of current and novel therapeutic approaches. J Natl Cancer Inst. (2019) 111:1131–41. doi: 10.1093/jnci/djz093, PMID: 31322663 PMC6855933

[7] Gelsomino F Barbolini M Spallanzani A Pugliese G Cascinu S. The evolving role of microsatellite instability in colorectal cancer: A review. Cancer Treat Rev. (2016) 51:19–26. doi: 10.1016/j.ctrv.2016.10.005, PMID: 27838401

[8] Hu H Kang L Zhang J Wu Z Wang H Huang M. Neoadjuvant PD-1 blockade with toripalimab, with or without celecoxib, in mismatch repair-deficient or microsatellite instability-high, locally advanced, colorectal cancer (PICC): a single-centre, parallel-group, non-comparative, randomised, phase 2 trial. Lancet Gastroenterol Hepatol. (2022) 7:38–48. doi: 10.1016/S2468-1253(21)00348-4, PMID: 34688374

[9] Cercek A Lumish M Sinopoli J Weiss J Shia J Lamendola-Essel M. PD-1 blockade in mismatch repair-deficient, locally advanced rectal cancer. N Engl J Med. (2022) 386:2363–76. doi: 10.1056/NEJMoa2201445, PMID: 35660797 PMC9492301

[10] Chalabi M Verschoor YL Tan PB Balduzzi S Van Lent AU Grootscholten C. Neoadjuvant immunotherapy in locally advanced mismatch repair-deficient colon cancer. N Engl J Med. (2024) 390:1949–58. doi: 10.1056/NEJMoa2400634, PMID: 38838311

[11] Amin MB Greene FL Edge SB Compton CC Gershenwald JE Brookland RK. The Eighth Edition AJCC Cancer Staging Manual: Continuing to build a bridge from a population-based to a more “personalized” approach to cancer staging. CA Cancer J Clin. (2017) 67:93–9. doi: 10.3322/caac.21388, PMID: 28094848

[12] Benson AB Venook AP Al-Hawary MM Cederquist L Chen YJ Ciombor KK. Rectal cancer, version 2.2018, NCCN clinical practice guidelines in oncology. J Natl Compr Canc Netw. (2018) 16:874–901. doi: 10.6004/jnccn.2018.0061, PMID: 30006429 PMC10203817

[13] Eisenhauer EA Therasse P Bogaerts J Schwartz LH Sargent D Ford R. New response evaluation criteria in solid tumours: revised RECIST guideline (version 1.1). Eur J Cancer. (2009) 45:228–47. doi: 10.1016/j.ejca.2008.10.026, PMID: 19097774

[14] Clavien PA Barkun J de Oliveira ML Vauthey JN Dindo D Schulick RD. The Clavien-Dindo classification of surgical complications: five-year experience. Ann Surg. (2009) 250:187–96. doi: 10.1097/SLA.0b013e3181b13ca2, PMID: 19638912

[15] Chalabi M Fanchi LF Dijkstra KK Van den Berg JG Aalbers AG Sikorska K. Neoadjuvant immunotherapy leads to pathological responses in MMR-proficient and MMR-deficient early-stage colon cancers. Nat Med. (2020) 26:566–76. doi: 10.1038/s41591-020-0805-8, PMID: 32251400

[16] Lenz HJ Van Cutsem E Luisa Limon M Wong KYM Hendlisz A Aglietta M. First-line nivolumab plus low-dose ipilimumab for microsatellite instability-high/mismatch repair-deficient metastatic colorectal cancer: the phase II checkMate 142 study. J Clin Oncol. (2022) 40:161–70. doi: 10.1200/JCO.21.01015, PMID: 34637336

[17] Fan A Wang B Wang X Nie Y Fan D Zhao X. Immunotherapy in colorectal cancer: current achievements and future perspective. Int J Biol Sci. (2021) 17:3837–49. doi: 10.7150/ijbs.64077, PMID: 34671202 PMC8495390

[18] Tumeh PC Harview CL Yearley JH Shintaku IP Taylor EJ Robert L. PD-1 blockade induces responses by inhibiting adaptive immune resistance. Nature. (2014) 515:568–71. doi: 10.1038/nature13954, PMID: 25428505 PMC4246418

[19] Rooney MS Shukla SA Wu CJ Getz G Hacohen N. Molecular and genetic properties of tumors associated with local immune cytolytic activity. Cell. (2015) 160:48–61. doi: 10.1016/j.cell.2014.12.033, PMID: 25594174 PMC4856474

[20] Wang F Zhao Q Wang YN Jin Y He MM Liu ZX. Evaluation of POLE and POLD1 mutations as biomarkers for immunotherapy outcomes across multiple cancer types. JAMA Oncol. (2019) 5:1504–6. doi: 10.1001/jamaoncol.2019.2963, PMID: 31415061 PMC6696731

[21] Domingo E Freeman-Mills L Rayner E Glaire M Briggs S Vermeulen L. Somatic POLE proofreading domain mutation, immune response, and prognosis in colorectal cancer: a retrospective, pooled biomarker study. Lancet Gastroenterol Hepatol. (2016) 1:207–16. doi: 10.1016/S2468-1253(16)30014-0, PMID: 28404093

[22] Durando ML Menghani SV Baumann JL Robles DG Day TA Vaziri C. Four-year disease-free remission in a patient with POLE mutation-associated colorectal cancer treated using anti-PD-1 therapy. J Natl Compr Canc Netw. (2022) 20:218–23. doi: 10.6004/jnccn.2021.7115, PMID: 35276675

[23] Benson AB Venook AP Al-Hawary MM Azad N Chen YJ Ciombor KK. NCCN Clinical Practice Guidelines in Oncology (NCCN Guidelines). Colon Carcinoma. Version 1.2024. (2024). Available online at: https://www.nccn.org/guidelines.

[24] Boutros C Tarhini A Routier E Lambotte O Ladurie FL Carbonnel F. Safety profiles of anti-CTLA-4 and anti-PD-1 antibodies alone and in combination. Nat Rev Clin Oncol. (2016) 13:473–86. doi: 10.1038/nrclinonc.2016.58, PMID: 27141885

[25] Khoja L Day D Wei-Wu Chen T Siu LL Hansen AR. Tumour- and class-specific patterns of immune-related adverse events of immune checkpoint inhibitors: a systematic review. Ann Oncol. (2017) 28:2377–85. doi: 10.1093/annonc/mdx286, PMID: 28945858

[26] Diaz Jr LA Shiu KK Kim TW Jensen BV Jensen LH Punt C. Pembrolizumab versus chemotherapy for microsatellite instability-high or mismatch repair-deficient metastatic colorectal cancer (KEYNOTE-177): final analysis of a randomised, open-label, phase 3 study. Lancet Oncol. (2022) 23:659–70. doi: 10.1016/S1470-2045(22)00197-8, PMID: 35427471 PMC9533375

[27] Chen G Jin Y Guan WL Zhang RX Xiao WW Cai PQ. Neoadjuvant PD-1 blockade with sintilimab in mismatch-repair deficient, locally advanced rectal cancer: an open-label, single-centre phase 2 study. Lancet Gastroenterol Hepatol. (2023) 8:42231. doi: 10.1016/S2468-1253(22)00439-3, PMID: 36870360

[28] Cui H Cui JX Wang YN Cao B Deng H Zhang KC. Could neoadjuvant chemotherapy increase postoperative complication risk of laparoscopic total gastrectomy? A mono-institutional propensity score-matched study in China. World J Gastrointest Surg. (2021) 13:429–42. doi: 10.4240/wjgs.v13.i5.429, PMID: 34122733 PMC8167844

[29] Molenaar CJL Minnella EM Coca-Martinez M Ten Cate DWG Regis M Awasthi R. Effect of multimodal prehabilitation on reducing postoperative complications and enhancing functional capacity following colorectal cancer surgery: the PREHAB randomized clinical trial. JAMA Surg. (2023) 158:572–81. doi: 10.1001/jamasurg.2023.0198, PMID: 36988937 PMC10061316

